# Supplementary material for: SnapFISH-IMPUTE: an imputation method for multiplexed DNA FISH data
Source: Commun Biol. 2024 Jul 9;7:834. doi: 10.1038/s42003-024-06428-7 (PMC11233503; doi:10.1038/s42003-024-06428-7)
Supplement: Supplementary file 1 — Supplementary Information [file 42003_2024_6428_MOESM1_ESM.pdf]

SnapFISH-IMPUTE: an imputation method for multiplexed DNA FISH data

Hongyu Yu[1,2], Daiqing Wu[3], Shreya Mishra[4], Guning Shen[5,6], Huaigu Sun[7], Ming Hu[4,*], and Yun Li[5,7,8,*]

[1]Department of Statistics, University of Wisconsin-Madison, Madison, WI, USA
[2]Department of Biochemistry, University of Wisconsin-Madison, Madison, WI, USA
[3]Department of Mathematics, Sun Yat-Sen University, Guangzhou, Guangdong, China
[4]Department of Quantitative Health Sciences, Lerner Research Institute, Cleveland Clinic Foundation, Cleveland, OH, USA
[5]Department of Computer Science, University of North Carolina, Chapel Hill, NC, USA
[6]Department of Biology, University of North Carolina, Chapel Hill, NC, USA
[7]Department of Genetics, University of North Carolina, Chapel Hill, NC, USA
[8]Department of Biostatistics, University of North Carolina, Chapel Hill, NC, USA
[*]yun_li@med.unc.edu; hum@ccf.org

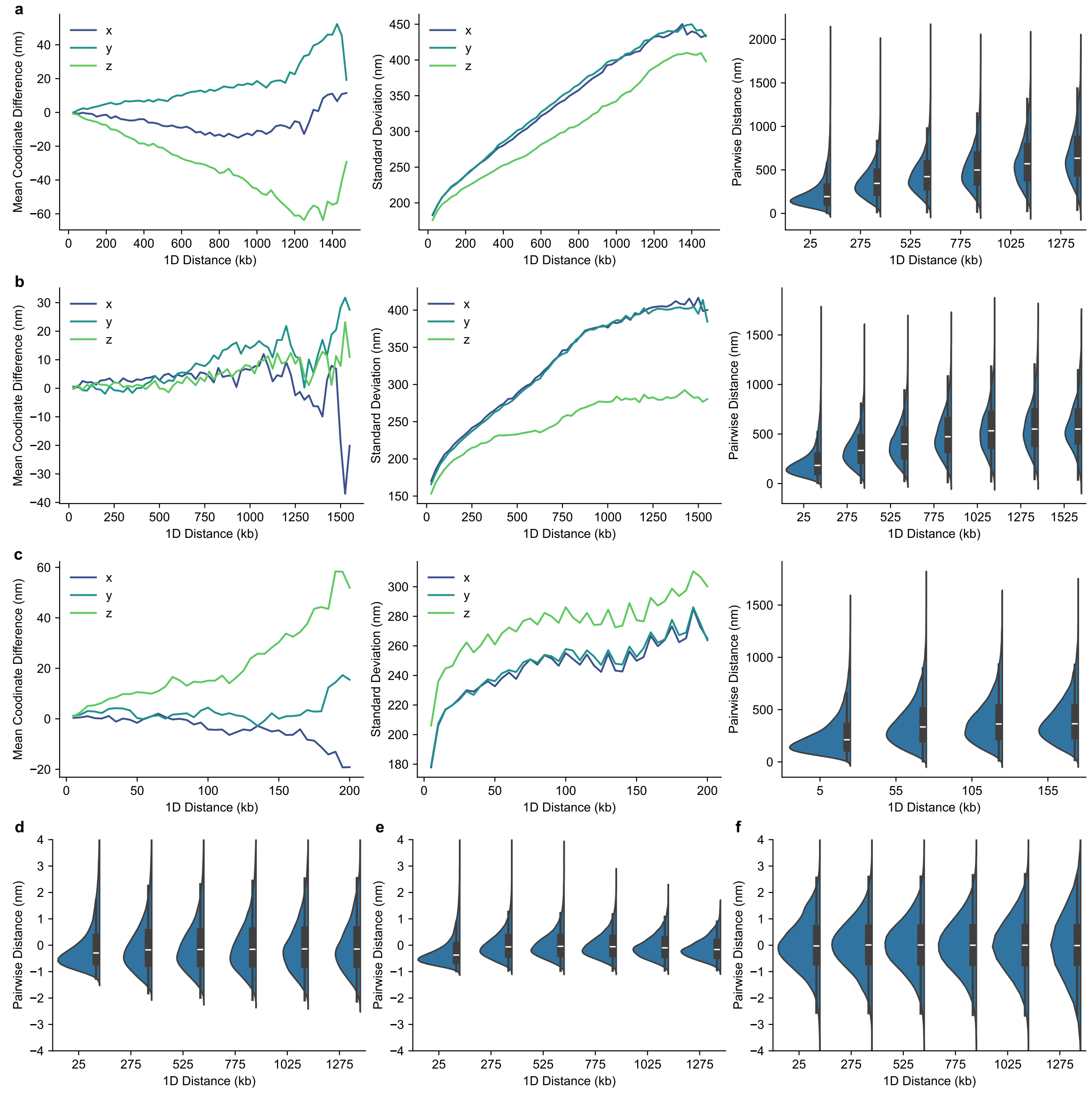

Figure S1: **3D coordinates from different resolutions and imaging protocols have distinctive characteristics.** **a** Imaging region 1 from the 25kb subset of the DNA seqFISH+ mESCs dataset. **b** Imaging region 2 from the 25kb subset of the DNA seqFISH+ mESCs dataset. **c** The 129 allele from the 5kb chromatin tracing dataset. Each row: the average distance in each axis between locus pairs with the same 1D genomic distance, the standard deviation in each axis between locus pairs with the same 1D genomic distance, and the Euclidean distances between locus pairs with the same 1D genomic distance. **d** z-score normalized pairwise distances. **e** GLM normalized pairwise distances. **f** Two-stage normalized pairwise distances.

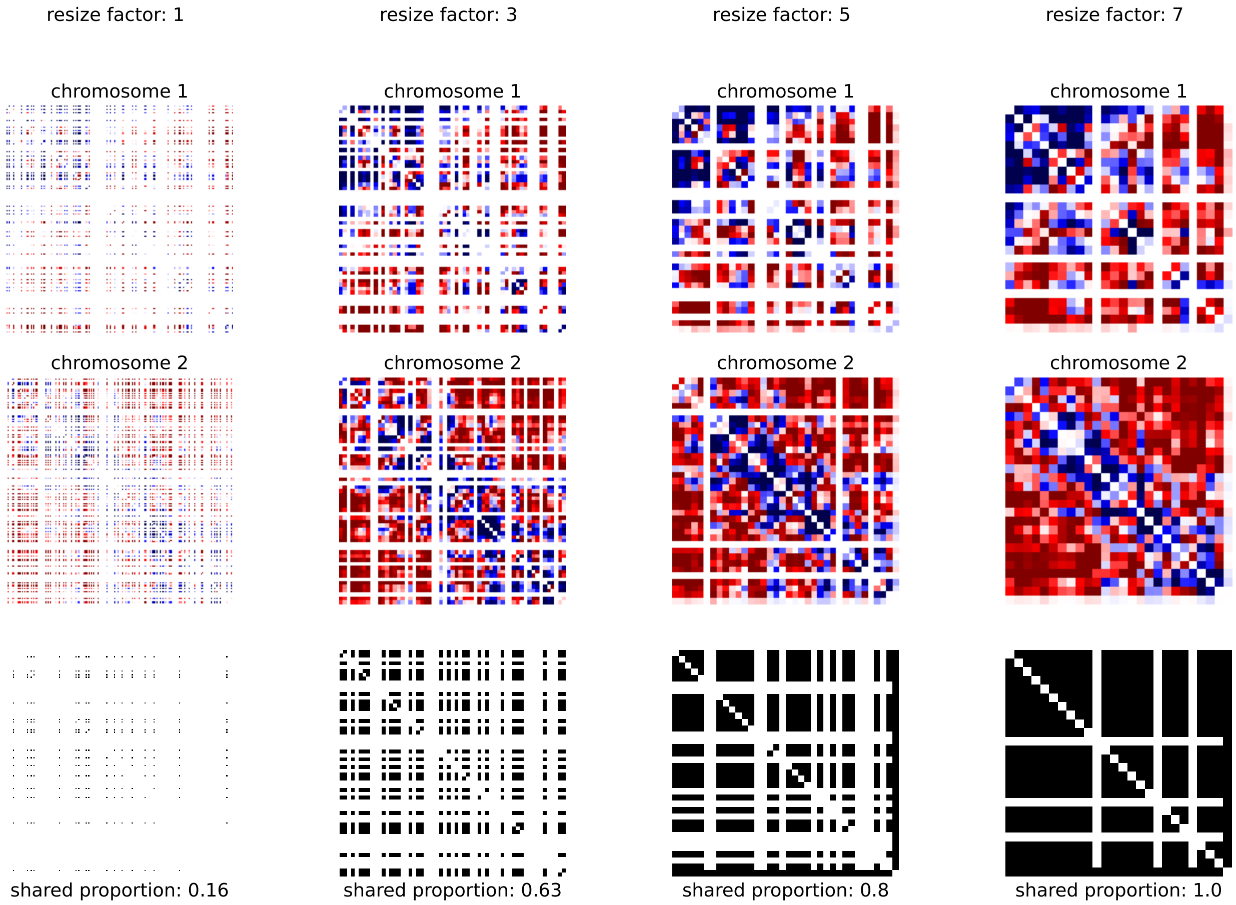

Figure S2: **More overlapping values after resizing.** The first row and the second row show the pairwise distance matrices of two cells under different resizing ratios. The last row shows the shared available entries between the two cells under different resizing ratios.

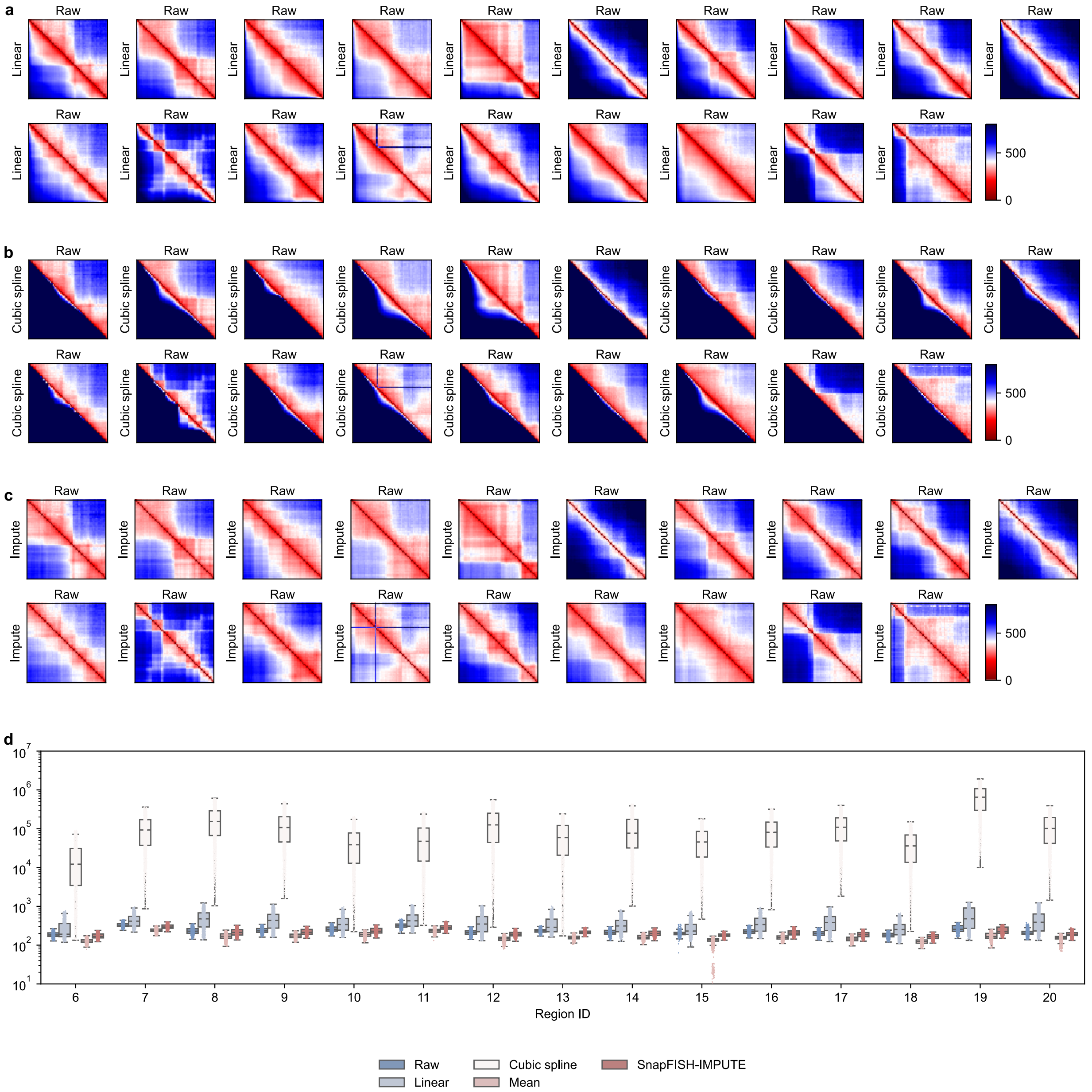

Figure S3: **The averages and the standard deviations of distance matrices from the 25kb subset of the DNA seqFISH+ mESCs dataset.** **a-c** Average pairwise distance matrices calculated from linear imputation, cubic spline imputation, and the proposed imputation method. Region 2 to 20 (autosome 2 to 19 and X cell) are shown. **d** The standard deviation of each entry in the distance matrix. Region 6 to 20 are shown. Box plot shows the first quartile, the median, the third quartile, and the min and max (excluding outliers) of the data (n=1770 possible pairs).

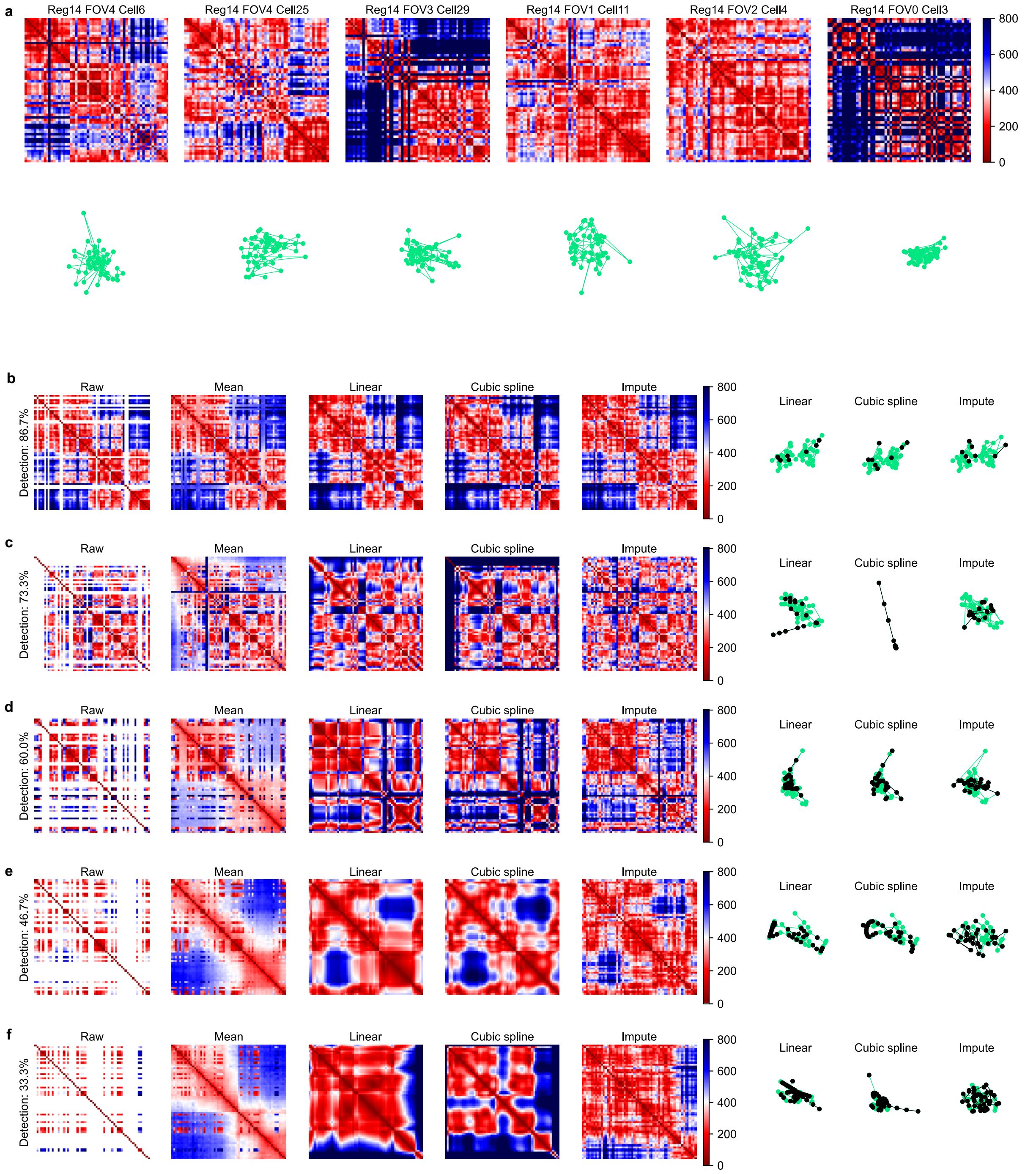

Figure S4: **Single cell examples.** **a** Cells with no missing loci are selected from the 25kb subset of the DNA seqFISH+ mESCs dataset (Reg: imaging region; FOV: field of view; Cell: cell ID). The first row is the pairwise distance matrices, and the second row is the 3D conformations. **b**-**f** Cells with decreasing detection efficiencies. The distance matrices of the raw data and the imputed data are shown. The last three plots in each line are the 3D conformations from the linear imputation result, cubic spline imputation result, and the imputation result from our method. Green dots are observed loci, and black dots are imputed loci.

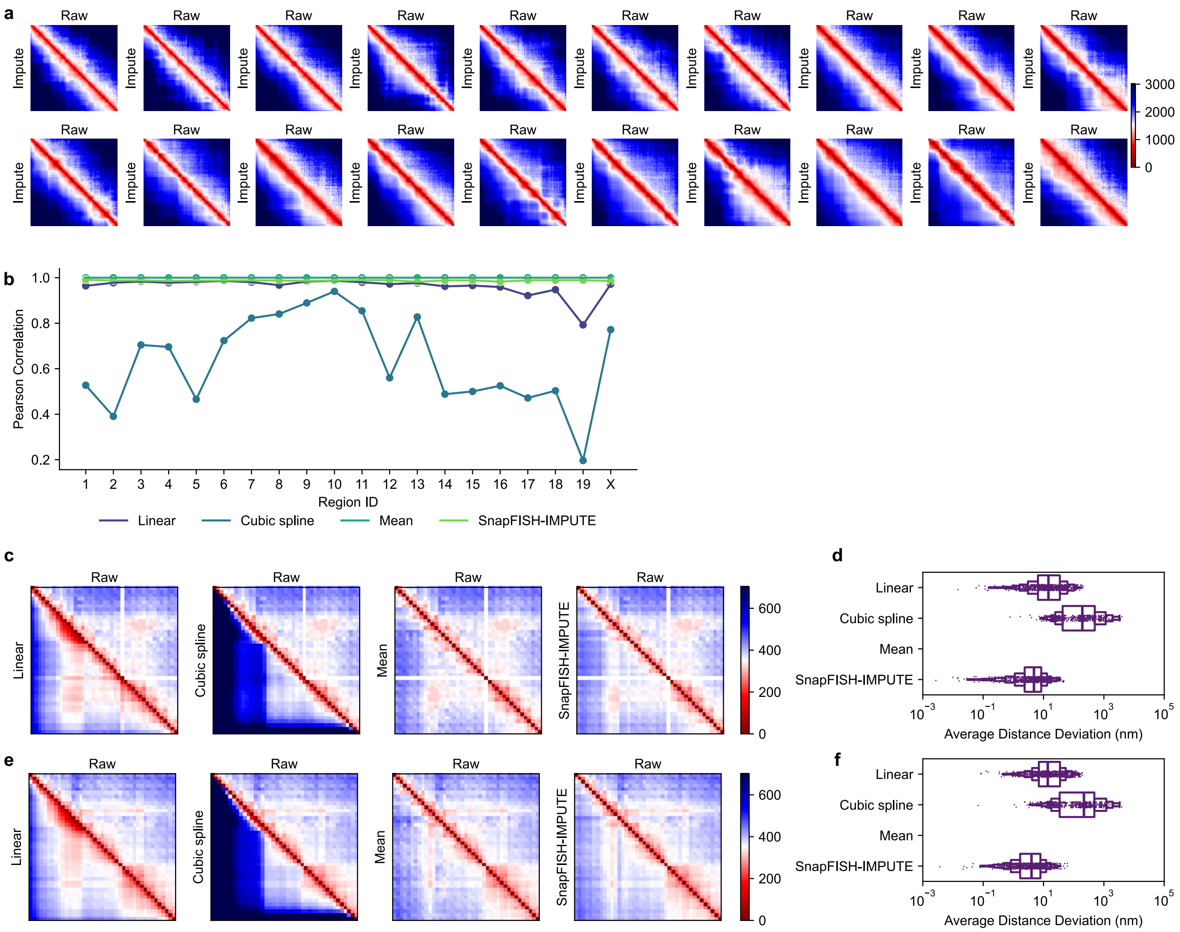

Figure S5: **The proposed imputation method generalizes well to other imaging datasets with different resolutions and imaging protocols.** **a** The average distance matrix of each imaging region in the 1Mb subset of the DNA seqFISH+ mESCs dataset. The upper triangle is the raw distance matrix, and the lower triangle is the imputed distance matrix. **b** Pearson correlations between the raw average distance matrix and the average distance matrix calculated from different imputation methods. **c** Average pairwise distance matrix of the 129 allele from the 5kb chromatin tracing dataset. **d** The absolute difference between the upper triangle and the lower triangle in part **c** (n=780). **e** Average pairwise distance matrix of the CAST allele from the 5kb chromatin tracing dataset. **f** The absolute difference between the upper triangle and the lower triangle in part **e** (n=820).

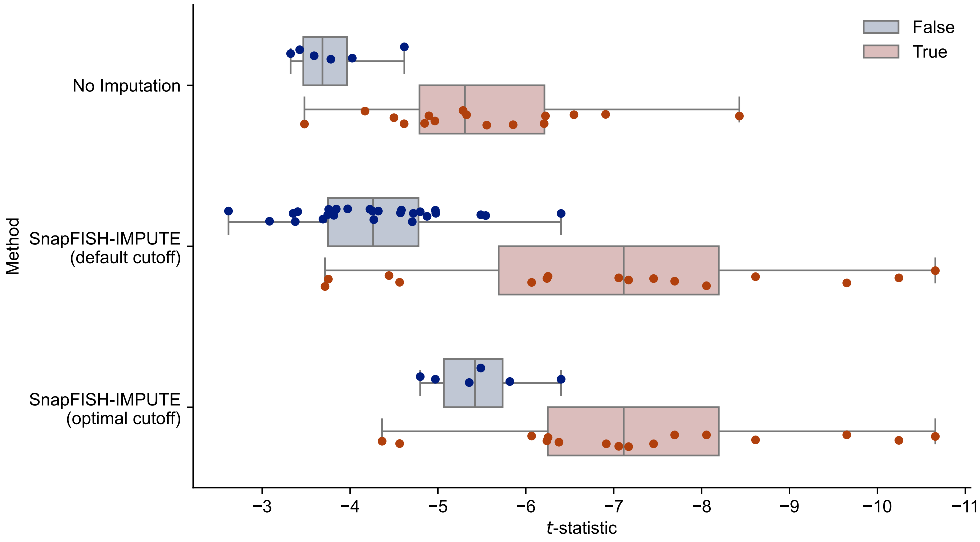

Figure S6: **The t-statistics of loops called by SnapFISH using FitHiC2 output as the ground truth.** This is the same as figure 3c except that the loops are called by FitHiC2 instead of HiCCUPS. Box plot shows the first quartile, the median, the third quartile, and the min and max (excluding outliers) of the data (n=6, 16 loops for no imputation; n=26, 16 loops for SnapFISH-IMPUTE with the default cutoff; n=6, 16 loops for SnapFISH-IMPUTE with the optimal cutoff).

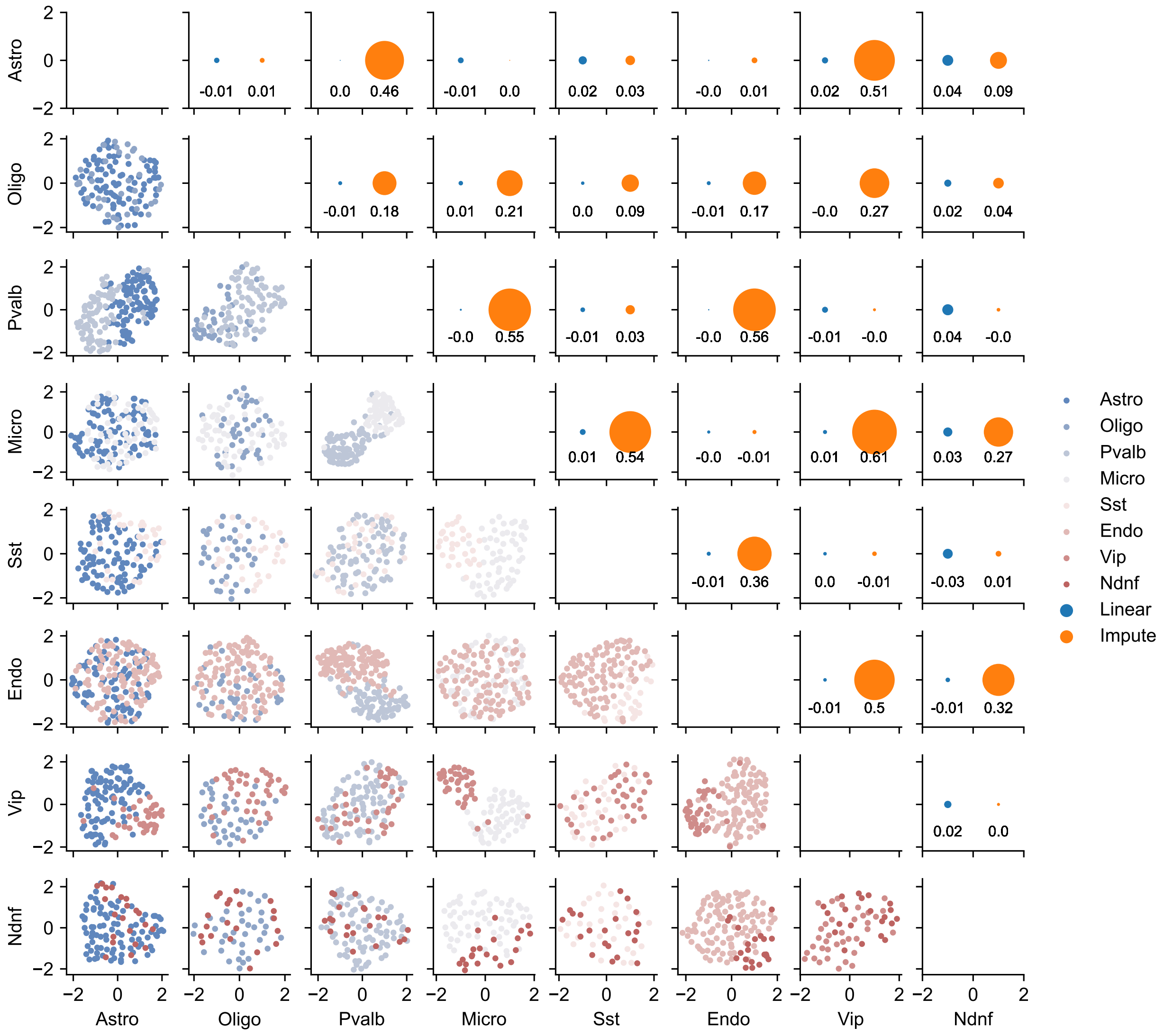

Figure S7: **Imputation allows cell type clustering using 3D chromatin conformations.** Eight major cell types of mouse brain cells are shown. Excitatory neurons are excluded because of their large numbers compared to other cell types. The lower left plots are pairwise UMAP plots between any two cell types. The dimension of the original data is first reduced to twenty with PCA before performing UMAP. The upper right plots are the adjusted mutual information scores between the clustered data and the ground truth from mRNA data. Both linear imputation result and SnapFISH-IMPUTE result are shown.
