# Supplementary material for: SnapFISH-IMPUTE: an imputation method for multiplexed DNA FISH data
Source: Commun Biol. 2024 Jul 9;7:834. doi: 10.1038/s42003-024-06428-7 (PMC11233503; doi:10.1038/s42003-024-06428-7)
Supplement: Supplementary file 2 — Description of Additional Supplementary Materials [file 42003_2024_6428_MOESM2_ESM.docx]

**Description of Additional Supplementary Files**

**File name:** Supplementary Data 1

**Description:** the source data behind the graphs in the paper
